# Swietenine Alleviates Nonalcoholic Fatty Liver Disease in Diabetic Mice via Lipogenesis Inhibition and Antioxidant Mechanisms

**DOI:** 10.3390/antiox12030595

**Published:** 2023-02-27

**Authors:** Kit-Kay Mak, Shiming Zhang, Jestin Chellian, Zulkefeli Mohd, Ola Epemolu, Albena T. Dinkova-Kostova, Madhu Katyayani Balijepalli, Mallikarjuna Rao Pichika

**Affiliations:** 1 School of Postgraduate Studies, International Medical University, 126 Jalan Jalil Perkasa 19, Bukit Jalil, 57000 Kuala Lumpur, Malaysia; 2 Pharmaceutical Chemistry Department, School of Pharmacy, International Medical University, 126 Jalan Jalil Perkasa 19, Bukit Jalil, 57000 Kuala Lumpur, Malaysia; 3 Centre of Excellence for Bioactive Molecules and Drug Delivery, Institute for Research, Development & Innovation (IRDI), International Medical University, 126 Jalan Jalil Perkasa 19, Bukit Jalil, 57000 Kuala Lumpur, Malaysia; 4 Department of Life Sciences, School of Pharmacy, International Medical University (IMU), Bukit Jalil, 57000 Kuala Lumpur, Malaysia; 5 Principal Research Scientist-In Vitro/In Vivo DMPK, Charles River Laboratories Edinburgh Ltd., Tranent, East Lothian EH33 2NE, Scotland, UK; 6 Division of Cellular and Systems Medicine, School of Medicine, University of Dundee, Dundee DD1 4HN, UK; 7 Departments of Medicine and Pharmacology and Molecular Sciences, Johns Hopkins University, Baltimore, MA 21218, USA; 8 Department of Pharmacology, Faculty of Medicine, Bioscience & Nursing, MAHSA University, Jln SP 2, Bandar Saujana Putra, Jenjarom 42610, Selangor, Malaysia

**Keywords:** swietenine, swietenia macrophylla, diabetes, NAFLD, lipogenesis, NRF2, oxidative stress

## Abstract

Our previous studies have reported the effect of swietenine (a major bioactive component of Swietenia macrophylla seeds) in reversing and potentiating the effect of metformin in hyperglycemia and hyperlipidaemia in diabetic rats. Moreover, we reported that the anti-inflammatory effect of swietenine is mediated via the activation of nuclear factor erythroid 2-related factor 2 (Nrf2). This study evaluated the effect of swietenine and its mechanisms in nonalcoholic fatty liver disease (NAFLD) in high-fat diet/streptozotocin-induced diabetic mice. The effect was assessed by determining blood biochemical parameters (glucose, cholesterol, triglycerides, alanine transaminase (ALT), asparate transaminase (AST), alkaline phosphatase (ALP), glutathione (GSH), total antioxidant capacity (TAC), and malondialdehyde (MDA)) and liver biochemical parameters (liver index, cholesterol, and triglycerides). Hepatic lipid accumulation (initial causative factor in NAFLD) was determined by oil-O-red staining. Gene expression (qPCR) and immunohistochemical studies were performed to elucidate the mechanism of swietenine’s effect in NAFLD. The critical regulators (genes and proteins) involved in lipogenesis (ACLY, ACC1, FASN, SREBP1c, and ChREBPβ) and oxidative stress (Nrf2, NQO-1 and HO-1) pathways were determined. In mice fed with a high-fat diet followed by streptozotocin injection, the liver cholesterol, triglycerides, and lipids were elevated. These increases were reversed by the oral administration of swietenine, 80 mg/kg body weight, on alternate days for eight weeks. Gene expression and immunohistochemical studies showed that swietenine reversed the elevated levels of crucial enzymes of lipogenesis (ACLY, ACC1 and FASN) and their master transcription factors (SREBP1c and ChREBPβ). Furthermore, swietenine activated the Nrf2 antioxidant defense mechanism, as evidenced by the upregulated levels of Nrf2, NQO-1, and HO-1. It is concluded that swietenine shows beneficial effects in diabetes-induced NAFLD via inhibiting lipogenesis and activating the Nrf2 pathway.

## 1. Introduction

The liver plays a central and crucial role in regulating glucose metabolism [1], fatty acid metabolism [2], and oxidative stress [3]. Dysregulated hepatic antioxidant status and metabolism of fatty acids and glucose cause liver damage, leading to nonalcoholic fatty liver disease (NAFLD) [4,5,6,7,8,9,10,11,12]. The development of fatty liver disease without excessive alcohol consumption is called NAFLD, one of the most common liver diseases accounting for nearly 30% of liver disease worldwide [13,14,15,16,17,18,19,20,21]. NAFLD is a metabolic disease in which elevated plasma triglycerides and low-density lipoproteins are the hallmarks [22]. Patients with NAFLD have significantly increased mortality because of both hepatic (such as cirrhosis and hepatocellular carcinoma) and extrahepatic complications (metabolic syndrome, cardiovascular disease, and malignancy) [23]. The development of NAFLD strongly correlates with diabetes, as > 90% of obese patients with diabetes also have NAFLD [24]. The continued production of triglycerides by the liver and the concomitant failure to suppress glucose production lead to hyperglycaemia, hyperlipidaemia, and hepatic steatosis [25]. Triglycerides are synthesized in the liver via the esterification of fatty acids, which have different origins. Hepatic fatty acids can result from local synthesis from acetyl CoA, but they can also result from direct uptake from plasma [26]. Abnormal fat deposition, increased hepatic enzyme activities, hepatic fibrosis, and liver cirrhosis are representative liver abnormalities in NAFLD associated with diabetes [25]. 

In diabetes, glycolysis provides carbons for de novo lipogenesis, which is under the control of various enzymes [27,28,29]; Adenosine triphosphate citrate lysase (ACLY), Acetyl-CoA carboxylase 1 (ACC1), Fatty acid synthase (FASN), carbohydrate response element binding protein β (ChREBPβ), and sterol regulatory element binding protein 1c (SREBP1c). Multiple studies have shown the protective role of nuclear factor erythroid 2-related factor 2 (Nrf2), a master regulator of antioxidative status, in the etiology and progression of NAFLD [30,31,32,33]. Currently, antidiabetic and antihyperlipidemic drugs are being used to prevent the symptoms of NAFLD [34]. No definitive pharmacological treatment has been approved for the treatment of NAFLD [35]. Streptozotocin is reported to cause liver toxicity, with similar characteristics observed in NAFLD [36]. 

There is evidence that ethnopharmacology, the scientific exploration of traditional medicinal plants, has provided lead compounds for treating various diseases [37]. In the literature, many plants and their bioactive compounds are reported to possess excellent antidiabetic [38] and antihepatotoxic [39] activities. *Swietenia macrophylla* seeds are used in traditional medicine to treat diabetes, and the bioactive compound responsible for the antidiabetic activity is swietenine, a nortetratriterpenoid [40]. Many scientific studies have demonstrated the antidiabetic activities of *S. macrophylla* [41,42,43,44]. We have recently reported that swietenine activates Nrf2 [40] and potentiates metformin’s antidiabetic activity [45]. This study aims to investigate the effect of swietenine on crucial regulators of de novo lipogenesis, fatty acid oxidation, and oxidative stress, which play a vital role in the development of NAFLD in streptozotocin-induced diabetic mice. 

## 2. Materials and Methods

### 2.1. Animal Studies

Our previous studies reported that swietenine at 40 mg/kg body weight showed a significant reversal of hyperglycaemia and hyperlipidaemia in streptozotocin-induced diabetic rats [45]. C57BL/6J mice were used as the experimental animals in this study. The dose was calculated by applying a correction factor (K_m_) [46,47]. The dose of swietenine used in this study was 80 mg/kg body weight. The experimental protocol was approved by International Medical University (IMU)-Joint Committee on Research and Ethics (Ref: IMU R143/2014). The animals were taken care of following IMU’s animal care guidelines. Male C57BL/6J mice (weight range 18–22 g, 6 weeks of age) were purchased from University Putra Malaysia and acclimatized for 2 weeks to the experimental conditions by housing in IMU animal house (maintained at 12/12 h light/dark cycle, temperature 25 ± 3 °C, 45 ± 5% humidity) with ad libitum access to a standard pellet diet (Altromin 1324, Altromin GmbH, Lage, Germany) and drinking water. Type2 diabetes was induced by feeding the mice with a high-fat pelleted diet (70% energy from 42% fat, Altromin C 1090-70, Altromin GmbH, Lage, Germany) for 3 weeks, followed by a single intraperitoneal injection (60 mg/kg body weight, dissolved in freshly prepared 0.05 M citrate buffer, pH 4.5) of streptozotocin (Sigma Chemical Co., St. Louis, MO, USA). Blood glucose levels were measured one week after the streptozotocin injection. The mice whose fasting (overnight) blood glucose levels were greater than 150 mg/dL were divided into diabetic control (Group D, 10 mice fed with a high-fat diet for 8 weeks) and treatment group (Group S, 10 mice fed with a high-fat diet and oral administration of swietenine 80 mg/kg body weight on alternate days for 8 weeks). The normal control mice (Group N, 10 mice) were fed with a standard pellet diet for 8 weeks. At the end of the experiment, the mice were anaesthetised with ketamine and sacrificed by cervical dislocation. Blood was collected by cardiac puncture, and liver organs were excised for histological, gene expression, and immunohistochemistry studies.

### 2.2. Biochemical Studies

The serum biochemical parameters: glucose, cholesterol, triglycerides, alkaline phosphatase (ALP), asparate transaminase (AST), and alanine transaminase (ALT) were performed on the ‘Siemens Dimension Xpand Plus integrated chemistry system’ with software version 10.1.2 (Siemens Healthcare Diagnostics, Inc.) as described in our previous paper [45]. Glutathione (GSH), malondialdehyde (MDA), and total antioxidant capacity (TAC) in serum were determined using respective assay kits from Sigma-Aldrich (Reduced Glutathione (GSH) Assay Kit (MAK364), Lipid Peroxidation (MDA) Assay Kit (MAK085) and Total Antioxidant Capacity Assay Kit (MAK187) following the manufacturer’s instructions. The mice’ body and liver weights were determined to calculate the liver index $\left(=\frac{liver\;weight}{body\;weight}\times 100\%\right).$ The cholesterol and triglycerides levels in liver homogenates were also determined.

### 2.3. Histological Studies

The histology of the liver tissues was examined using Oil-O-Red (Abcam, #ab223796, USA) staining techniques. The Oil O Red stains lipid droplets bright red and is routinely used to determine lipid accumulation in the tissues [48]. The frozen tissues were cut into 5-μm thick sections, fixed with 4% paraformaldehyde at 4 °C for 30 min, and then washed with phosphate buffer saline and 60% isopropanol. The liver sections were stained with Oil Red O stain for 1 h at room temperature and then washed with 60% isopropanol, followed by PBS. The tissue slides were cleared with xylene and were mounted with a coverslip using a DPX mounting medium (histological grade, # 06522, Sigma Aldrich, Saint Louis, MO, USA). The histology of the tissue sections was observed under Nikon Eclipse 80i Microscope (magnification, ×400). The results were analyzed in three randomly selected fields of view in each section using the panoramic scanner (3DHISTECH Ltd., Hungary). The average densities of collagen fibres (Masson’s trichome staining) and fat droplets (Oil O Red staining) were calculated. Oil O Red staining was carried out in the dark.

### 2.4. Real-Time Quantitative PCR (RT-qPCR) Assay

The liver tissues were homogenized in liquid nitrogen, and total RNA was extracted (n = 10 per group) with QIAzol^®^ (Qiagen, Austin, TX, USA) according to the manufacturer’s protocol. The concentration and purity of RNA were measured using an Ultra-Micro UV Visible Spectrophotometer (TECAN Infinite M200 Pro). RNA samples (100 ng) were dissolved in DNase/RNase-free water (Thermofisher, Waltham, MA, USA). ReverTra Ace^®^ qPCR RT Master Mix Kit was used to synthesize first strand cDNAs according to the manufacturer’s instructions as follows: 37 °C for 15 min, 50 °C for 5 min, 98 °C for 5 min. The qPCR primers used in the present study (obtained from Integrated DNA Technologies, USA) were presented in Table 1. qPCR was performed using THUNDERBIRDTM Next SYBR ^®^ qPCR Mix (Toyobo STC CO., LTD. Osaka, Japan) and an Applied CFX96 Touch Real-Time PCR Detection System (Bio Rad Laboratories, Inc., California, CA, USA). The PCR cycling conditions were 95 °C for 1 min, 40 cycles of 95 °C for 15 s, 60 °C for 25 s, and 72 °C for 45 s. The reference gene, β-actin, was used as a reference gene for the normalization of target gene expression, and the relative expression of genes was determined using the 2^−ΔΔCt^ method [49].

### 2.5. Immunohistochemical Studies

The paraffin-embedded liver tissues were cut into 4-μm thick sections using a rotary microtome and allowed to float on warm water. The immunohistochemistry (IHC) protocol published in the literature elsewhere was followed [50,51]. The tissue sections were (1) transferred onto IHC microscope slides (FLEX, Agilent), (2) dried at room temperature, (3) deparaffinised with xylene, (4) hydrated with 100%, 95%, 70%, and 50% ethanol sequentially, (5) incubated at room temperature in 3% hydrogen peroxide (#H1009, Sigma Aldrich, Saint Louis, MO, USA) solution in methanol at room temperature for 10 min, (6) rinsed with PBS, (7) placed in staining dishes (Thermo Fisher Scientific, Waltham, MA, USA), (8) incubated with citrate buffer (10 mM, pH 6.0) at 95 °C for 10 min, (9) cooled down to room temperature, (10) washed with PBS, (11) incubated the with blocking buffer (100 μL, 10% foetal bovine serum (Tico, Europe) in PBS) at 25 °C for 1 h, (12) incubated with the primary antibody (antibodies were purchased from Abcam), at 25 °C for 1 h, (13) washed with PBS, (14) incubated with biotinylated secondary antibody (Abcam (ab64256) at 25 °C for 1 h, (15) washed with PBS, (16) incubated in the dark with Streptavidin-Horseradish Peroxidase (HRP, # ab7403, Abcam, Cambridge, UK) conjugates at 25 °C for 1 h, (17) washed with PBS, (18) supplemented with freshly prepared 2,4′-dihydroxyacetophenone dioxygenase (DAB) substrate kit (Abcam (ab64238), (18) washed with PBS, (19) counterstained with haematoxylin for 5 min, (20) rinsed with distilled water, (21) dehydrated with 95% and 100% ethanol, (22) dipped in xylene, and (23) mounted with a coverslip using a mounting medium (Abcam (ab64320), USA). The slides were observed under the microscope (Nikon Eclipse Ts2-FL, Nikon Instruments Inc. New York, NY, USA)), and the colour intensity was quantified (6 images from random areas of interest at 400X from each tissue) using the software ImageJ Fiji (version 1.2; WS Rasband, National Institute of Health, Bethesda, MD, USA) following the protocol reported in the literature [52,53,54].

### 2.6. Statistical Analysis

Results are presented as mean ± standard deviation (SD) of six readings. The difference between the two groups was determined using one-way ANOVA followed by Dunnett’s multiple comparisons tests. GraphPad Prism version 9.0.1 for Windows, GraphPad Software, San Diego, CA, USA, was used for performing the statistical analysis. *p* < 0.05 is considered statistically significant.

## 3. Results

### 3.1. Effect of Swietenine on Biochemcial Parameters

**Figure 1:**
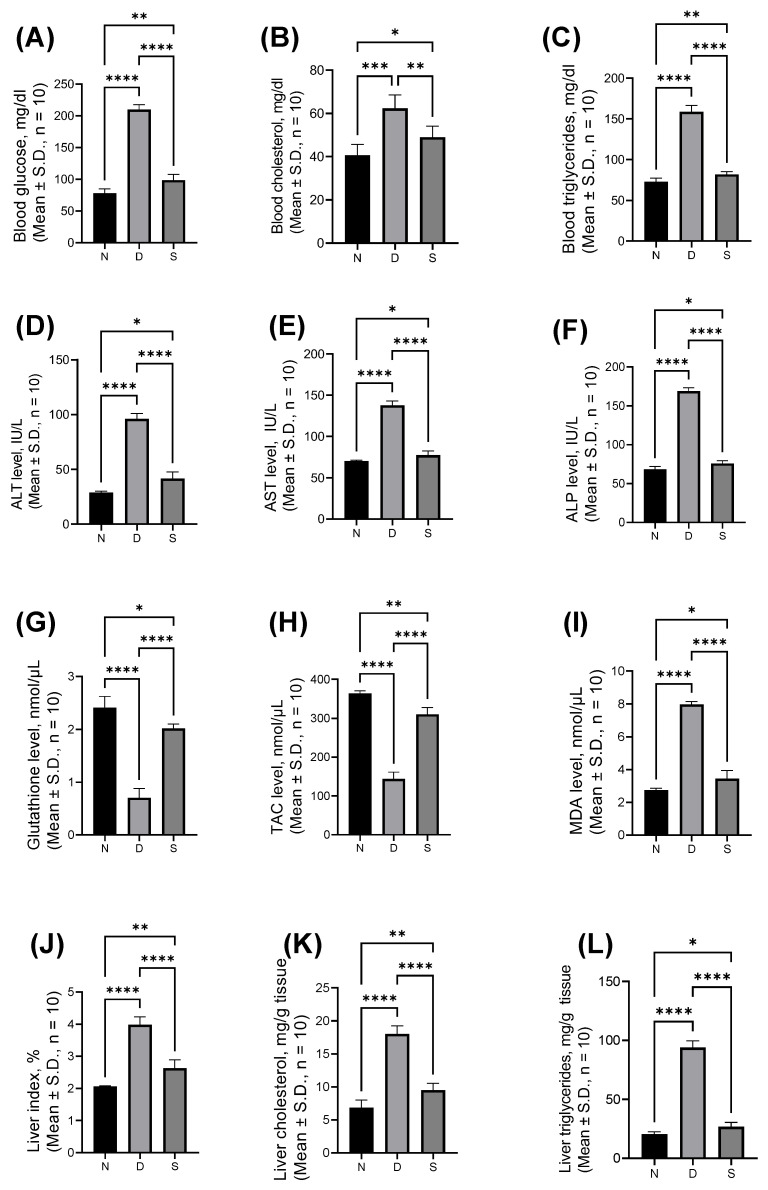
The biochemical parameters of Swietenine effect in serum and liver samples in (**A**) blood glucose, (**B**) blood cholesterol, (**C**) blood triglycerides, (**D**) alanine aminotransferase, ALT levels, (**E**) aspartate aminotransferase, AST levels, (**F**) alkaline phosphatase, ALP levels, (**G**) glutathione level, (**H**) total antioxidant capacity, TAC levels, (**I**) malondialdehyde, MDA level, (**J**) liver index, (**K**) liver cholesterol and (**L**) liver triglycerides. N, normal group; D, diabetic mice group; S, treatment with swietenine (80 mg/kg) group. ns: Not significant; * *p* < 0.05, ** *p* < 0.01, *** *p* < 0.001, **** *p* < 0.0001.

The effect of Swietnine on serum and liver biochemical parameters is shown in Figure 1. The blood glucose, cholesterol, and triglyceride levels were elevated in diabetic mice (209.9 ± 7.71, 62.33 ± 6.22, and 158.7 ± 7.94 mg/dL, respectively) compared to control mice (78.36 ± 6.63, 40.67 ± 5.01, and 72.83 ± 4.31 mg/dL, respectively). Swieteine reversed the elevated blood glucose, cholesterol, and triglycerides levels to 98.67 ± 9.11, 49.00 ± 5.06, and 81.83 ± 3.55 mg/dL, respectively. The ALT, AST, and ALP levels in normal control mice were 28.83 ± 1.17, 70.08 ± 1.20, and 68.25 ± 3.95 IU/L, respectively. These levels were increased to 96.00 ± 4.94, 137.80 ± 5.19, and 169.00 ± 4.43 IU/L, respectively, in diabetic mice, and the increased levels were reversed by swietenine to 41.67 ± 6.05, 77.33 ± 5.24, and 76.00 ± 3.58 IU/L, respectively. The levels of antioxidant markers (GSH and TAC) in blood were lowered in diabetic mice (0.71 ± 0.18 and 143.90 ± 17.36 nmol/μL, respectively) compared to normal control mice (2.41 ± 0.21 and 364.10 ± 6.74 nmol/μL, respectively). Treatment with swietenine reversed the elevated levels of GSH and TAC to 2.02 ± 0.08 and 310.50 ± 17.65 nmol/μL, respectively. The level of oxidative stress marker, MDA, was increased in diabetic mice (7.98 ± 2.74 nmol/μL compared to 2.75 ± 0.11 nmol/μL), which was reversed upon treatment with swietenine (3.45 ± 0.49 nmol/μL). The liver index (%) was 2.06 ± 0.01 in normal mice, which was increased to 3.98 ± 0.25 in diabetic mice, and upon treatment with swietenine, it was reversed to 2.63 ± 0.26. The liver cholesterol and triglycerides levels were elevated in diabetic mice (18.00 ± 1.27 and 93.83 ± 5.71 mg/dL, respectively) from normal mice, in which the levels were 6.83 ± 1.17 and 20.50 ± 1.87 mg/dL, respectively. Swietenine treatment reversed the levels to 9.50 ± 1.05 and 26.83 ± 3.54 mg/dL, respectively.

### 3.2. Effect of Swietenine on Fat Accumulation

**Figure 2:**
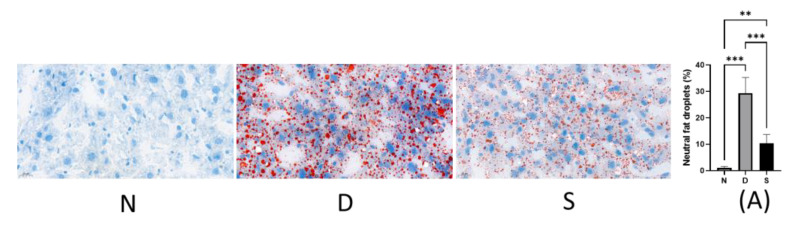
Representative images of Oil red O stained sections of the liver sections. (N) Normal rat’s liver section, (D) Diabetic rat’s liver section, (S) Swietenine (80 mg/kg) treated diabetic rat’s liver section, (A) Quantification of neutral lipids. ** *p* < 0.01, *** *p* < 0.001.

NAFLD is the common cause of chronic liver disease under diabetic conditions. The effect of swietenine treatment on hepatic lipid homeostasis was assessed by quantifying the lipid content using Oil Red O staining. In diabetic mice (D), the neutral fat droplets were significantly increased (from 0.99% ± 0.24 in normal mice to 29.29% ± 5.94 in diabetic mice). Swietenine treatment reversed the elevated levels of neutral fat droplets (from 29.29% ± 5.94 in diabetic mice to 10.30 ± 1.38). The results are shown in Figure 2, and these findings suggest that swietenine regulates hepatic lipid homeostasis in diabetes and helps prevent NAFLD development.

### 3.3. Effect of Swietenine on Lipogenesis Enzymes and Regulators

Three enzymes, ATP citrate lysase (ACLY), acetyl CoA carboxylase isoform 1 (ACC1), and fatty acid synthase (FASN), are the key enzymes involved in the de novo lipogenesis [55]. The first step in lipogenesis is the conversion of citrate to acetyl-CoA, catalysed by ACLY. Then, ACC1 carboxylates acetyl-CoA to malonyl-CoA, from which fatty acids are synthesized by FASN [29]. ACLY is a crucial lipogenic enzyme that catalyzes an ATP-consuming reaction to generate acetyl-CoA from citrate, and acetyl-CoA is the critical building block for de novo lipogenesis [56]. ACC is a rate-limiting enzyme for de novo lipogenesis that catalyzes the synthesis of malonyl-CoA, a substrate for fatty acid synthesis and the regulator of fatty acid oxidation [57]. FASN catalyzes the de novo lipogenesis by synthesizing long-chain fatty acids from acetyl-CoA and malonyl-CoA [58]. Sterol regulatory element-binding protein-1c (SREPB1C) is a master transcription regulator of the enzymes involved in de novo lipogenesis. Its expression in diabetes is elevated in response to increased insulin levels [59]. Carbohydrate-responsive element-binding protein (ChREBPβ) is another transcription regulator of the enzymes involved in de novo lipogenesis. Its expression in diabetes is elevated in response to increased glucose levels [59].

**Figure 3:**
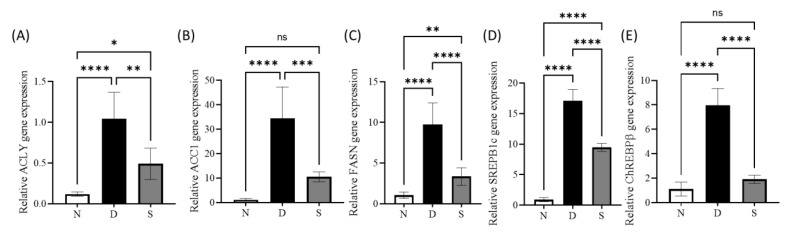
Effect of Swietenine on key enzymes involved in de novo lipogenesis (**A**) ACLY, (**B**) ACC1 and (**C**) FASN, and two transcriptional lipogenesis regulatory genes ((**D**) SREPB1c and (**E**) ChREBPβ). It is observed that these genes are significantly upregulated in diabetic mice, but the effect was significantly reversed in the treatment group with swietenine (80 mg/kg). The treatment with swietenine resulted in a non-significant ACCA and ChREBPβ difference in the levels compared to their respective normal group. N, normal group; D, diabetic mice group; S, treatment with swietenine (80 mg/kg) group. ns: Not significant; * *p* < 0.05, ** *p* < 0.01, *** *p* < 0.001, **** *p* < 0.0001.

qPCR and immunohistochemistry studies were carried out to study the effect of swietenine on genes, transcription factors, and proteins involved in de novo lipogenesis. The gene expression study revealed the upregulation genes of three key enzymes (ACLY, ACC1, and FASN) in diabetic mice, and swietenine treatment reversed the elevated levels (Figure 3A–C). The levels of ACLY, ACC1, and FASN in diabetic mice were 1.04 ± 0.13, 34.48 ± 5.22, and 9.77 ± 0.76, respectively, whereas these levers were reduced to 0.49 ± 0.08, 10.47 ± 0.82, and 3.35 ± 0.31, respectively, in swietenine-treated diabetic mice. In addition, the transcriptional lipogenesis regulatory genes (SREPB1c and ChREBPβ, Figure 3D,E) were upregulated in diabetic mice, and these levels were reversed upon treatment with swietenine.

**Figure 4:**
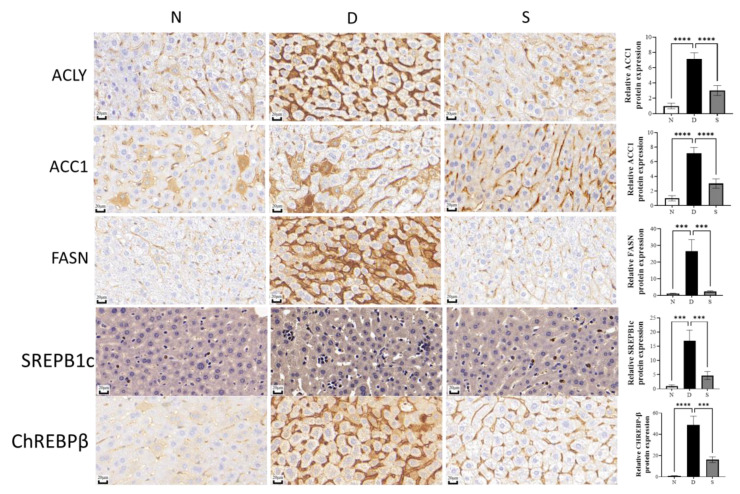
Immunohistochemistry studies of the effect of Swietenine on ACLY, ACC1, FASN, SREPB1c and ChREBPβ and their respective protein expression. N, normal group; D, diabetic mice group; S, treatment with swietenine group (80 mg/kg). In the diabetic group, the levels of the key enzymes involved in de novo lipogenesis (ACYL, ACCA and FASN) levels and the transcriptional regulators (SREPB1c and ChREBPβ) increased significantly. However, the swietenine (80 mg/kg) treatment group significantly reversed the effects. ns: Not significant; *** *p* < 0.001, **** *p* < 0.0001.

The levels of SREPB1c and ChREBPβ in diabetic mice were 17.07 ± 0.54 and 7.96 ± 0.56, respectively, whereas their levers in swietenine-treated diabetic mice were 9.46 ± 0.19 and 1.90 ± 0.14, respectively. Immunohistochemical studies revealed the upregulation of all three key enzymes (ACLY, ACC1, and FASN) involved in the de novo lipogenesis in diabetic mice, and swietenine treatment reversed the elevated levels (Figure 4). ACLY, ACC1 and FASN in diabetic mice were 3.88 ± 0.24, 7.15 ± 0.33, and 26.59 ± 2.82, respectively, whereas the levels in swietenine-treated diabetic mice were 1.13 ± 0.06, 3.02 ± 0.26, and 2.28 ± 0.19, respectively. In addition, immunohistochemical studies also revealed the upregulation of two transcriptional regulators (SREBP1c and ChREBPβ) in diabetic mice, which are reversed upon treatment with swietenine (Figure 4). SREBP1c and ChREBPβ levels in diabetic mice were 16.91 ± 1.53 and 48.84 ± 3.38, respectively, whereas the levels in swietenine-treated diabetic mice were 4.69 ± 0.57 and 16.22 ± 1.12, respectively.

### 3.4. Effect of Swietenine on Crucial Regulators of Oxidative Stress

**Figure 5:**
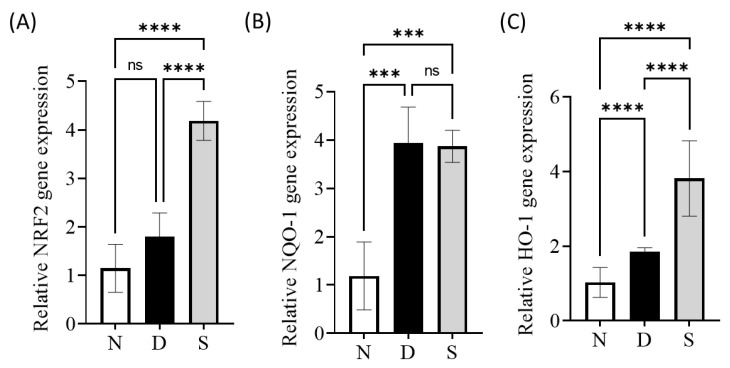
Effect of Swietenine on Nrf2, NQO-1 and HO-1 gene expression with respect to the reference gene. N, normal group; D, diabetic mice group; S, treatment with swietenine (80 mg/kg) group. (**A**) The levels of NRF2 in the normal and diabetic groups were insignificant. However, there was a 20-fold increase after the diabetic mice were treated with swietenine. (**B**) NQO1 and (**C**) HO-1 levels slightly increased in the diabetic group compared to those in normal mice. The treatment with Swietenine upregulated the NQO1 and HO-1 by close to 20-fold. ns: Not significant;, *** *p* < 0.001, **** *p* < 0.0001.

The transcription factor Nrf2 is a master regulator of adaptive response to oxidative stress and is reported to play a vital role in alleviating NAFLD [60]. The Nrf2 transcriptional targets include NADPH quinone oxidoreductase 1 (NQO-1) and heme oxygenase-1 (HO-1), which play roles in antioxidative responses that counteract the effects of oxidative stress [61]. Several reports have shown that the pharmacological activation of Nrf2 in the liver reduced liver lipid levels [62,63,64]. Our previous studies (in vitro) reported that swietenine activates Nrf2 [40]. In this study, qPCR and immunohistochemical analyses were carried out to determine the expression of Nrf2, NQO-1, and HO-1 genes and proteins. Gene expression studies revealed that the mRNA levels for all these three genes were elevated in diabetic mice, and their levels were further increased upon treatment with swietenine (Figure 5). The mRNA levels for Nrf2, NQO-1, and HO-1 in diabetic mice were 1.80 ± 0.48, 3.94 ± 0.74, and 1.85 ± 0.12, respectively, whereas their levels in swietenine-treated diabetic mice were 4.19 ± 0.40, 3.87 ± 0.39, and 3.82 ± 1.01, respectively.

**Figure 6:**
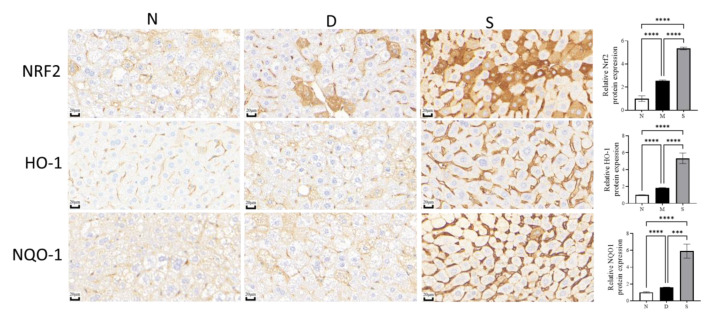
Immunohistochemistry studies of the effect of Swietenine on NRF2, NQO1 and HO-1. N, normal group; D, diabetic mice group; S, treatment with swietenine group (80 mg/kg). In the diabetic group, these proteins were upregulated, whereas the swietenine treatment (80 mg/kg) significantly upregulated the proteins further. ns: Not significant; *** *p* < 0.001, **** *p* < 0.0001.

Immunohistochemical studies revealed the levels of Nrf2, NQO-1, and HO-1 in diabetic mice were upregulated (fold increase compared to control, Figure 6) by 2.53 ± 0.03, 1.59 ± 0.01, and 1.82 ± 0.02 whereas swietenine treatment further upregulated (fold increase compared to control) by 5.34 ± 0.05, 5.91 ± 0.34, and 5.39 ± 0.25, respectively.

## 4. Discussion

The association between NAFLD and diabetes is bidirectional. Diabetes causes NAFLD and leads to nonalcoholic steatohepatitis (NASH), liver cirrhosis and liver cancer. In contrast, NAFLD increases the risk of diabetes development [65]. De novo lipogenesis and oxidative stress are the characteristics of NAFLD [66,67]. The key enzymes involved in lipogenesis are ACLY, ACC1 and FASN, and these enzymes are regulated by two master transcription regulators, SREBP1c and ChREPBβ [68]. In addition, lipogenesis and diabetes are also associated with oxidative stress, which is a crucial factor in the progression of NAFLD to NASH and HCC [69]. Nrf2 is a master regulator of the antioxidant defense system against the toxic effects of endogenous and exogenous oxidants. Many studies have highlighted the benefits of Nrf2 activators in diabetes and NAFLD [30]. In our previous work, we reported the antihyperglycaemic, antihyperlipidaemic, and antioxidant effects of swietenine and its synergistic effects with metformin in diabetic rats [45]. Moreover, we have reported that the anti-inflammatory effect of swietenine is mediated via Nrf2 activation [40]. Based on the above-said findings, we hypothesized that swietenine (at the dose of 80 mg/kg b.w.) exhibits beneficial effects in diabetes-induced NAFLD via reversing the 1) upregulated expression of critical enzymes involved in de novo lipogenesis (ACLY, ACC1, and FASN) and their transcription factors (SREBP1c and ChREPBβ), and 2) activation of the Nrf2 pathway. Feeding C57BL/6J mice with HFD followed by streptozotocin injection exhibited the symptoms of NAFLD; 1) elevated liver cholesterol, and triglycerides levels, 2) increased lipid accumulation, and 3) increased ratio of liver to body weight (liver index). Oral administration of swietenine (80 mg/kg b.w.) on alternate days for eight weeks reversed the symptoms of NAFLD in the liver. Gene expression and immunohistochemical studies have shown that swietenine down-regulates the critical enzymes (ACLY, ACC1, and FASN) of lipogenesis, the master regulators (SREBP1c and ChREPBβ) of lipogenesis enzymes, and critical regulators of antioxidant defense mechanism (Nrf2, NQO-1, and HO-1). Notably, we have previously shown that genetic or pharmacological Nrf2 activation downregulates fatty acid synthesis and upregulates fatty acid oxidation [70,71,72]. Moreover, our previous studies reported that swietenine was stable in liver microsomes [40], suggesting that the bioactivity observed in this study is because of the swietenine itself.

*Swietenia macrophylla* seeds are used in folk medicine to maintain health and treat various diseases such as diabetes, hypertension, inflammation, sexual dysfunction etc. [73]. Various herbal supplements (such as coffee, oil, capsules, extract, etc.) containing *S. macrophylla* were developed and are available in the market. Some supplements have received a patent and approval from the Ministry of Health Malaysia https://news.utm.my/ms/2021/04/goswiet-after-7-years-of-research-swietenia-mahagoni-attracted-diabetic-consumer/ accessed on 7 February 2023). Many researchers in Malaysia are researching *S. macrophylla* seeds to explore their medicinal value. Our survey found that the general public suffering from diabetes consumes *S. macrophylla* seeds and prescription medicines together. Our previous study showed that swietenine potentiates the effect of metformin in diabetic rats [45]. In continuation of our previous studies [40,45,74] on the most bioactive compound of *S. macrophylla* seeds, swietenine, we have investigated the activity of swietenine in diabetes-induced NAFLD. Although our studies have shown promising bioactivity, there are limitations in the study design (because of time and financial constraints): (1) NAFLD is a multifactorial disease [75], and there is no single physiologically relevant animal model [76]. Thus, future studies must be carried out to confirm the bioactivity of swietenine in other animal models. (2) We could not be able to perform the pharmacokinetics studies in this study, and pharmacokinetics is the critical element to determine the dose and dosage of swietenine for its consumption. Thus, future studies must be carried to determine the pharmacokinetics of swietenine. (3) The general public consume *S. macrophylla* seeds either as-it-is or in powder form or capsule form and the seeds contain many bioactive compounds and nutrients in addition to swietenine (the bioactivity could also be contributed by those compounds and nutrients). Thus, future studies should be focused to determine the effect of whole seeds powder (we have attempted to determine the bioactivity of the seeds powder but we were unsuccessful because of challenges associated with administration of seeds powder to animals). (4) Since the people consume the seeds and did not report toxic effects, in our opinion, it is not advisable to assume its safety without confirming its safe use scientifically. Thus, detailed safety studies should be carried out to confirm its safe use for therapeutic interventions.

## 5. Conclusions

From the results of this study, it is concluded that swietenine has shown encouraging beneficial effects in a diabetes-associated NAFLD animal model. Swietenine reversed the hyperglycaemia-induced lipogenesis and oxidative stress. Switenien reversed the elevated levels of blood glucose, cholesterol, and triglycerides in blood and liver, hepatic function markers (ALT, AST, and ALP) in blood, and regulated the oxidative stress markers (glutathione, total antioxidant capacity, and malonaldehyde). The lipogenesis inhibitory activity of swietenine was confirmed using histological studies (Oil-O-Red staining) and gene and protein (ACLY, ACC1, FASN, SREBP1c and ChREPBβ) expression studies. The ability of swietenine to upregulate the master regulator of oxidative stress (Nrf2) is also confirmed using gene and protein expression (NRF2, HO-1 and NQO1) studies. Thus, biochemical, gene expression and protein expression studies have demonstrated the bioactivity of swietenine in diabetes-induced NAFLD. However, future studies should be conducted to determine the bioactivity and pharmacokinetics of swietenine in other NAFLD animal models to confirm the activity of swietenine. The work presented in this paper is the first study reporting the effect of swietenine on NAFLD in diabetic mice and the mechanisms involved.

## Figures and Tables

**Table 1:** The list of primers used in this study.

Gene Code	Gene Name	Sequence (5’-3’)	Accession Number
β-actin	Beta-actin	F: CGGTTCCGATGCCCTGAGGCTCTTR: CGTCACACTTCATGATGGAATTGA	NM_007393.5
ACLY	ATP citrate lysase	F: CTCACACGGAAGCTCATCAAR: TCCAGCATTCCACCAGTATTC	NM_001199296.1
ACC1	Acetyl CoA carboxylase isoform 1	F: CCCGTGAGAACACAGAGATAAAR: CTGAGGTGGTTGAGTGTGTT	NM_133360.3
FASN	Fatty acid synthase	F: GGATGTCAACAAGCCCAAATACR: GAGGAGAAGGCCACAAAGTAG	NM_007988.3
SREPB1c	Sterol regulatory element-binding protein-1c	F: GGAGCCATGGATTGCACATTR: GGCCCGGGAAGTCACTGT	NM_001358314.1
ChREBPβ	Carbohydrate-responsive element-binding protein	F: CCATCTTTGGACCTCCCTTTAATR: GAAACAGACCGACATCTCTCATC	NM_001359237.1
NRF2	Nuclear factor erythroid 2-related factor 2	F: CAAGACTTGGGCCACTTAAAAGAC R: AGTAAGGCTTTCCATCCTCATCAC	NM_010902.5
HO-1	Heme oxygenase-1	F: GTGATGGAGCGTCCACAGC R: TTGGTGGCCTCCTTCAAGG	NM_010442.2
NQO-1	NADPH quinone oxidoreductase 1	F: AGCTGGAAGCTGCAGACCTG R: CCTTTCAGAATGGCTGGCA	NM_008706.5

## Data Availability

Not applicable.
